# Endonasal Dacryocystorhinostomy: Results With or Without Stenting

**DOI:** 10.7759/cureus.33470

**Published:** 2023-01-06

**Authors:** Swati V Maldhure, Prajakta S Golhar, Prasanna P Moon

**Affiliations:** 1 Department of Otolaryngology - Head and Neck Surgery, Datta Meghe Medical College, Datta Meghe Institute of Medical Sciences (DU), Nagpur, IND; 2 Department of Otolaryngology - Head and Neck Surgery, Jawaharlal Nehru Medical College, Datta Meghe Institute of Medical Sciences (DU), Wardha, IND

**Keywords:** stent, endonasal dcr, lacrimal system, silicon stent, dacrocystitis

## Abstract

Introduction

This is a comparative cross-sectional study done to compare the curative outcome of endonasal dacryocystorhinostomy (DCR) done with and without a silicon stent in patients with chronic dacryocystitis due to nasolacrimal duct obstruction.

Methods

This study was carried out in central India, involving 60 patients who were diagnosed with chronic dacryocystitis and underwent endonasal DCR (with zero-degree and 45-degree endoscopes from Olympus, with an Olympus camera and monitor) between October 2021 and September 2022. All patients were over the age of 18, with major exclusion criteria of previous DCR and sinonasal disease. All the surgeries were done by the same senior surgeon, in which 30 patients were stented (with a Prison silicone nasolacrimal duct stent) and 30 of them were non-stented. The follow-up duration for these was 12 months after the surgery.

Results

The outcome was evaluated at the end of three months, six months, and 12 months for both groups (patients with and without stents) using the Chi-square test. The success rate at the end of six months was 90% with stented patients and 93.3% with non-stented patients (p-value - 0.64); at six months, it was also the same (p-value - 0.64); and at the end of 12 months, it was 80% and 76.6% (p-value - 0.71) for patients with and without a stent, respectively. The final endoscopy at the end of 12 months showed 93.3% of patients who underwent stenting had a patent rhinostomy opening, and 90% of those who were not stented had a patent opening.

Conclusions

Our observational comparative study showed that patients with chronic dacryocystitis who underwent endonasal DCR with and without stenting had almost similar results. There was no significant difference in the outcome. So, we concluded that generally all the patients should be considered for endonasal DCR without a stent, except in special cases like revision endonasal DCR, lacrimal gland cysts, fistulas, and patients with sinonasal pathology, in whom silicon stents can be preferred.

## Introduction

Epiphora is described as an overflow of tears. The level of epiphora can range from mild trickling to a continuous, annoying overflow. Obstruction of the nasolacrimal duct (NLD) can be divided into primary and secondary forms. The primary blockage of NLD is caused by inflammation and fibrosis for no apparent reason [1,2]. Secondary NLD block may be due to disease, inflammatory reaction, neoplastic, traumatic, or mechanical obstruction. It has been seen that primary obstruction of the NLD is seen mostly in middle-aged and older women, the reason being that they have a smaller middle NLD and nasolacrimal fossa [1,3].

The best curative option for NLD obstruction is dacryocystorhinostomy. It was first described by Toti in 1904 [4,5]. Later, endonasal or endoscopic approaches gained popularity because of the disadvantages of an external approach, like bleeding, increased time during surgery, and an external scar [6-8]. The merits of endonasal dacryocystorhinostomy are no scar, quicker surgery, and minimal blood loss. It has been noted that with the use of endoscopes in otorhinolaryngological surgeries, the ease of surgery has increased with a decrease in morbidity, with significantly better results and a better quality of life post-surgery endoscopic endonasal dacryocystorhinostomy has evolved from functional endoscopic sinus surgery [9-11]. In 1989, McDonogh and Meiring described endoscopic transnasal DCR [12]. The merits of endonasal dacryocystorhinostomy are no scar, quicker surgery, and minimal blood loss. In recent years, there have been a number of modifications to the procedure of endonasal DCR, including the use of silicon stents and the use of lasers like argon, carbon dioxide, potassium titanyl phosphate, and yttrium aluminum garnet (YAG). The latest is a transcanalicular approach with a neodymium-doped YAG laser that has also been described [12-16]. Since the use of a silicon stent is the most common modification and is widely used, there have been many controversies regarding performing endonasal DCR with or without a stent [17,18].

This article attempts to subjectively assess the short-term and long-term success rates of endonasal DCR with or without stents on the basis of a modified five-point Likert scale, which is a subjective assessment score [1,19].

## Materials and methods

This is a cross-sectional, observational, comparative study. Here, we conducted a study at Datta Meghe Medical College and Shalinitai Meghe Hospital and Research Centre, Wanadongri, Nagpur, Maharashtra. The study was approved by the Institutional Research Board of Shalinitai Meghe Hospital and Research Centre, Wanadongri, Nagpur (IRB approval number DMMC(DU)/IEC/2021/20). It involved 60 patients who were diagnosed with chronic dacryocystitis and underwent endonasal dacryocystorhinostomy between October 2021 and September 2022. The age of the patients selected was 18 years and above. The exclusion criteria included those below the age of 18, any eyelid anomalies, previous dacryocystorhinostomies, and any sinonasal pathology like chronic sinusitis, sinonasal polyposis, etc. Out of the 60 patients, 30 underwent endonasal DCR with a stent, and 30 of them underwent endonasal DCR without a stent. The follow-up duration for these patients was 12 months after the surgery, during which they were required to visit the OPD (outpatient department) at three months, six months, and 12 months. They were assessed on the basis of a modified five-point Likert scale, which is a subjective assessment score.

Surgical technique

In this study, all the patients underwent endonasal dacryocystorhinostomy under general anesthesia, using zero-degree and 45-degree endoscopes (endoscope, 4 mm, 0 degree and 45 degrees, Olympus Winter and IBE GMBH, Hamburg, Germany, with camera and monitor, Olympus Visera Elite OTV-S200, Olympus Medical Systems Corp., Tokyo, Japan). They were cleaned and draped under all aseptic precautions. Nasal cavities were decongested with 1:100,000 adrenalin in a 4% xylocaine solution and the lateral wall on the side of the disease. A "C"-shaped incision was made on the lateral nasal wall anterior to the attachment of the middle turbinate. A posteriorly based mucoperiosteal flap is elevated over the maxillary and lacrimal bones. The respective bones were identified, and the site of the lacrimal sac was located. The thick bone over the frontal process of the maxilla was removed using Smith-Kerrison punch forceps. The lacrimal sac was identified. A linear, vertical incision was made in the sac using a sickle knife, and the sac was opened. The vertical incision was converted into a book-shaped incision. The patency and flow of the dacryocystorhinostomy were confirmed. The mucosal flap was refashioned and repositioned on the lateral wall. In patients who were selected for stenting (Prison nasolacrimal preloaded silicone stents), the free ends of the stents were taped externally on the vestibule for easy accessibility postoperatively.

Postoperatively, patients were prescribed antibiotics, anti-inflammatory drugs, and antihistaminics for five days, normal saline nose drops for seven days; and moxifloxacin and steroids eye drops for seven days. The patient was discharged the next day after surgery. The stent was removed after one month of surgery. The results of the surgery were assessed at three months, six months, and one year using a modified five-point Likert scale.

## Results

The results were measured as the resolution of signs and symptoms (subjective assessment/functional success) and a patent rhinostomy opening (objective assessment/anatomical success). The subjective assessment was done using a modified five-point Likert scale [16]. A successful outcome was indicated by either complete resolution (score 1), significant symptomatic improvement (score 2), or slight improvement (score 3). Unsuccessful outcomes included no improvement (score 4) or worsening of symptoms (score 5). The patients were assessed endoscopically for patency of the rhinostomy opening during their follow-up in the OPD. In patients with a successful outcome, the patency of the rhinostomy was confirmed using syringing.

The outcome was evaluated at the end of three months, six months, and 12 months for both groups (patients with and without stents) using the Chi-square test.

The success rate at the end of three months was 90% with stented patients and 93.3% with non-stented patients (p-value=0.64) as seen in Table 1.

**Table 1:** Subjective (functional) results of endoscopic dacryocystorhinostomy at three months

Modified Likert scale	With stent (n=30)	Without stent (n=30)	p-value
1 (No symptoms)	24	22	
2 (Significant improvement)	3	4	
3 (Slight improvement)	2	2	
4 (No improvement)	1	2	
5 (Worsening of symptoms)	0	0	
Results	27 (90%)	28 (93.3%)	0.6469

It was observed that the success rate was similar at the end of six months (p-value of 0.64) as depicted in Table 2.

**Table 2:** Subjective (functional) results of endoscopic dacryocystorhinostomy at six months

Modified Likert scale	With stent (n=30)	Without stent (n=30)	p-value
1 (No symptoms)	24	22	
2 (Significant improvement)	3	4	
3 (Slight improvement)	2	2	
4 (No improvement)	1	2	
5 (Worsening of symptoms)	0	0	
Results	27 (90%)	28 (93.3%)	0.6469

Table 3 shows the result at the end of 12 months, which shows the success rate as 80% and 76.6%, respectively, for patients with and without a stent (p-value of 0.71).

**Table 3:** Subjective (functional) results of endoscopic dacryocystorhinostomy at 12 months

Modified Likert scale	With stent (n=30)	Without stent (n=30)	p-value
1 (No symptoms)	22	21	
2 (Significant improvement)	2	2	
3 (Slight improvement)	4	4	
4 (No improvement)	2	3	
5 (Worsening of symptoms)	0	0	
Results	24 (80%)	23 (76%)	0.7107

The final endoscopy at the end of 12 months showed 93.3% of patients who underwent stenting had a patent rhinostomy opening, and 90% of those who were not stented had a patent opening, as seen in Table 4. (p-value= 0.64).

**Table 4:** Objective (anatomical) results of endoscopic dacryocystorhinostomy at 12 months

Status of rhinostomy	With stent (n=30)	Without stent (n=30)	p-value
Rhinostomy open	28	27	
Rhinostomy closed	2	3	
Results	28 (93.33%)	27 (90%)	0.6469

## Discussion

In cases of chronic dacryocystitis in which the patient presents with epiphora due to nasolacrimal duct obstruction, endonasal dacryocystorhinostomy is an effective surgery to cause relief of symptoms. Here, not only can we avoid an external incision but also find out causes of DCR failure like synechiae, an enlarged middle turbinate, and ethmoid sinus disease [20]. Stenting of the canal, i.e., endocanalicular stenting after a dacryocystorhinostomy, is basically to maintain the openings of the punctum and the patency of the canaliculi during the postoperative period. On the other hand, in some cases, granulation tissue formation in the canaliculi has been seen during the postoperative period due to the use of stents along with punctual erosion [21,22]. Some studies have shown dacryocystorhinostomy without a stent has a short operative period, no complications associated with a stent, and avoids the trouble caused to patients for stent removal. Hence, a lot of controversies have been associated with whether stenting with a silicon stent should be done for patients who have chronic dacryocystitis and need an endonasal dacryocystorhinostomy [23]. Here, we have done a study on patients who needed endonasal dacryocystorhinostomies to compare the success rate of the use of stents in them. We evaluated 60 patients, of whom 30 underwent endonasal DCR without a stent and 30 with a stent. The stent was removed after one month of surgery. The outcome was evaluated at the end of three months, six months, and 12 months for both groups (patients with and without stents) using the Chi-square test. The success rate at the end of three months was 90% with stented patients and 86.6% with non-stented patients (p-value=0.3); at six months, it was also the same (p-value=0.3); and at the end of 12 months, it was 80% and 76.6% (p-value=0.5) for patients with and without a stent, respectively. The final endoscopy at the end of 12 months showed that 93% of patients who underwent stenting had a patent rhinostomy opening, and 90% of those who were not stented had a patent opening. Success rates of the endoscopic technique have been reported at 82%-95%, with stents being removed from four to 24 weeks postoperatively [1,24-26]. There have been cases with a primary success rate of 83 percent for endoscopic DCR with a stent, and in 17% of cases, the rhinostomy opening was found to be obstructed by granulations or synechia formation [1,25]. Some cases also report a success rate of 92.6% for endoscopic DCR without a stent, with no patients with major complications reported [1,27]. As most of the studies have, our study also has certain limitations. There is always a learning curve for all operating surgeons, and even if the surgeon is highly experienced, difficulties cannot be ruled out. The sample size may be inadequate. The duration of follow-up is also short, which does matter in the inference as the results of certain surgeries tend to fade with time. It is also true that the observers were not blinded. And patients were not randomized.

## Conclusions

Our observational comparative study showed that patients with chronic dacryocystitis who underwent endonasal dacryocystorhinostomy with and without stenting had almost similar results. There was no significant difference in the outcome. The patients who underwent endonasal dacryocystorhinostomy without a stent did equally well clinically and functionally as those who underwent the same with a nasolacrimal silicon stent. So, as per our observation, we concluded that generally all the patients should be considered for endonasal DCR without a stent, except in special cases like revision endonasal DCR, lacrimal gland cyst, fistula, and patients with sinonasal pathology, in whom silicon stents can be preferred, so that there is a less invasive intervention dealing with the canaliculi and puncta of the eye, granting better results and fewer complications.
